# The mediating role of health awareness in the relationship between health information behavior and health outcomes among the older adults

**DOI:** 10.3389/fpubh.2025.1492472

**Published:** 2025-07-18

**Authors:** Hong Li, Li Shi, Ya-Long Xing

**Affiliations:** ^1^Guangzhou Huashang College, Guangzhou, China; ^2^Faculty of Innovation and Design, City University of Macau, Taipa, Macao SAR, China

**Keywords:** health awareness, health information behavior, health of the older adults, structural equation model, health belief model

## Abstract

**Background:**

With global population aging, older adults face challenges in obtaining, understanding, and applying health information, which are critical for effective health management. Drawing on the Health Belief Model (HBM), this study examines how health information acquisition, understanding, and application influence older adults’ health awareness and, in turn, their health outcomes, with health awareness as a mediator.

**Methods:**

A structured questionnaire measured three health information behaviors (acquisition, understanding, application), health awareness, and self-reported health outcomes in a representative sample of community-dwelling older adults. Structural Equation Modeling (SEM) tested direct and indirect pathways linking these variables.

**Results:**

SEM revealed significant direct effects of health information behaviors on awareness: acquisition (*β* = 0.88, *p* < 0.001), understanding (*β* = 0.83, *p* < 0.001), and application (*β* = 0.94, *p* < 0.001). Health awareness then strongly predicted health outcomes (*β* = 0.93, *p* < 0.001). In single-factor mediation models, awareness significantly mediated the effects of acquisition and application on outcomes but not understanding. In the full model, acquisition and application maintained significant paths to awareness (*β* = 0.88 and 0.94, respectively; *p* < 0.001), whereas understanding did not (*β* = 0.05, *p* = 0.188). Indirect effects via awareness were largest for acquisition (0.58, *p* < 0.001), followed by understanding (0.53, *p* < 0.01) and application (0.29, *p* < 0.001).

**Conclusion:**

While all three information behaviors enhance health awareness, application and acquisition are most influential. Health awareness significantly mediates how acquisition and application translate into improved health outcomes. Public health strategies should therefore emphasize not only access to and comprehension of health information but, critically, its practical application through tailored digital platforms and community education.

## 1 Introduction

With the rapid acceleration of global aging, particularly in China, the proportion of the older adult population is rising sharply, presenting society with unprecedented health challenges. According to the “China Aging Development Report,” by 2050, the population aged 65 and above in China is expected to account for approximately 30% of the total population (1). In this context, achieving “healthy aging” has become a crucial component of national strategy. The Chinese government, through its “Healthy China 2030” initiative, has explicitly advocated for the concept of “proactive health,” encouraging the older adults to enhance their quality of life through active health management. The development of digital technology has provided unprecedented convenience for the dissemination of health information. According to the 48th “Statistical Report on Internet Development in China” by the China Internet Network Information Center, as of June 2021, 28.0% of internet users were aged 50 and above, reflecting a 5.2 percentage point increase from June 2020 (2). However, despite the significant achievements in promoting digital health, the development of digital networks has also exacerbated the “digital divide” among the older adults, further widening the gap between those who are technologically affluent and those who are technologically disadvantaged. This issue has become even more pronounced in the wake of the COVID-19 pandemic, which has led to significant shifts in societal perceptions of disease and health, driving the formation and development of a broader public health awareness (3). Research by Cui et al. has found that the frequency of digital technology use has significantly improved the dietary, sleep, exercise, smoking, and drinking habits of the older adults in China, thereby improving their overall health (4).

Health awareness is a key factor driving the older adults to adopt healthy behaviors. As individuals age, they face increasingly complex health issues, with a significant rise in the incidence of chronic diseases. Enhancing health awareness among the older adults not only encourages them to participate more actively in health management (5), such as regular check-ups, proper medication use, and lifestyle adjustments, but also effectively prevents and controls the occurrence and progression of chronic diseases. Furthermore, increasing health awareness empowers the older adults to take a proactive approach to health challenges, enabling them to better understand their own health status, improve their sensitivity to health information, and make more autonomous and confident health decisions. However, the older adults face numerous challenges in the process of acquiring and applying health information, primarily due to cognitive decline, limited access to information channels, and varying levels of health literacy (6). Relevant studies have shown that, a lack of health awareness makes the older adults more likely to overlook early signs of health problems, missing opportunities for prevention and timely treatment, thereby worsening their health condition (7).

Against the backdrop of accelerating digital health and an aging society in China, the relationship between internet use and health outcomes among the older adults has become increasingly complex. Therefore, it is of significant theoretical and practical importance to explore how the acquisition, understanding, and application of health information affect the health awareness of the older adults and how this awareness further influences health outcomes.

### 1.1 Theoretical foundation

With the development of digital health research, many studies have focused on health behaviors themselves and their direct outcomes, such as disease prevention, health promotion, and medical effectiveness. However, the mechanisms by which health literacy and behavior among the older adults can be enhanced remain an area that requires further exploration. The study by Chen et al. focuses on health information technologies (8), such as health apps and online health resources among the older adult population, examining how these technologies help the older adults better acquire and understand health information. However, this research mainly focuses on the use of technological tools and does not deeply analyze how these technologies can indirectly improve health outcomes by influencing health awareness. In contrast, Sørensen et al.’s study emphasizes the direct impact of health literacy on health outcomes across different populations (9), but its emphasis remains on the overall influence of health literacy on outcomes, lacking detailed exploration of how information processing specifically impacts health awareness. Van der Heide et al. conducted a multilevel analysis to explore the role of health literacy in different health domains (such as prevention, treatment, and management) (10). While they mentioned the importance of understanding and applying information, their study focused on comparing different levels of health literacy rather than thoroughly examining how these processes shape health awareness.

This study focuses on the health information processing of the older adults and introduces health awareness as a mediating variable. This approach not only extends the Health Belief Model (HBM) but also addresses the often-overlooked issue of health awareness in empirical research. The widespread use of the internet and the development of digital health technologies have catalyzed a “practical” transformation in older adult health behaviors, for which the HBM provides a theoretical framework. The HBM emphasizes the roles of perceived threats, perceived benefits, and perceived barriers in shaping health-related behaviors. The internet has evolved from being a tool for knowledge acquisition to facilitating the “practice” of health behaviors among the older adults. The HBM explains this transformation by highlighting the role of perceived threats, benefits, and barriers in shaping health-related behaviors.

**Figure 1:**
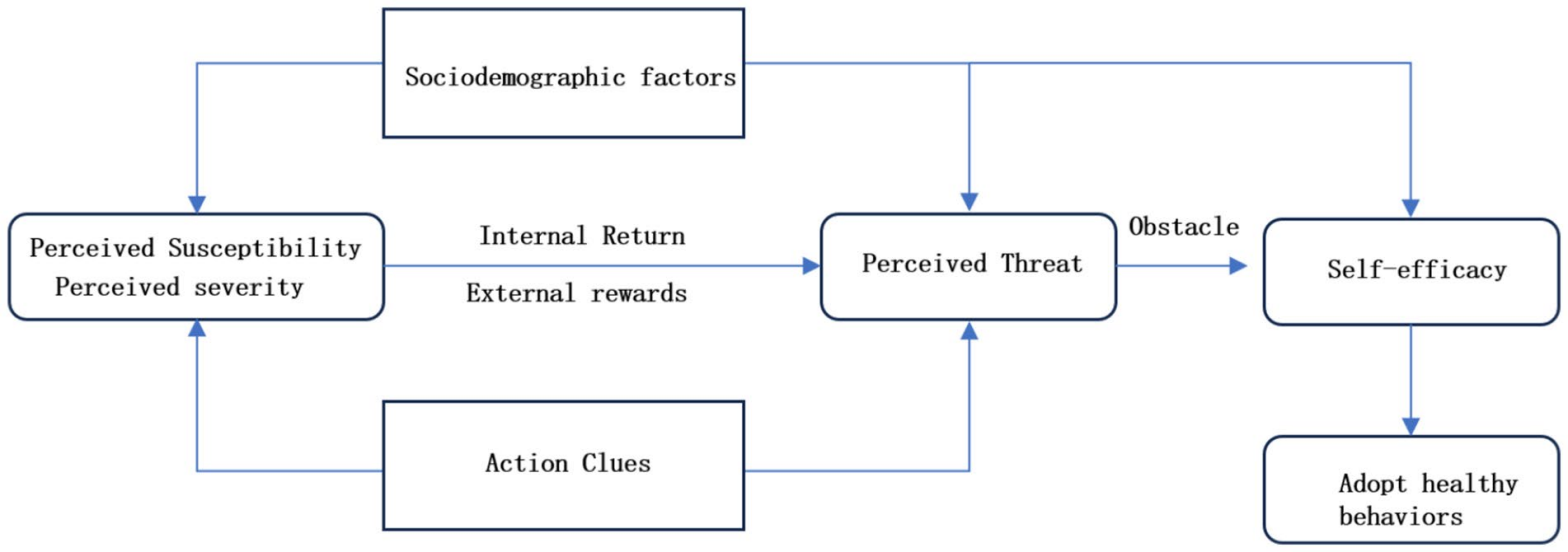
Theoretical framework of the health belief model (HBM).

Against this backdrop, the hypotheses and research model of this study are primarily based on the HBM (11), as shown in Figure 1. The HBM, proposed by Becker and his colleagues, is one of the earliest systematic theories to explore the determinants of individual health behaviors (12). This model emphasizes that perceived threats (such as the severity and susceptibility of diseases), as well as perceived benefits and barriers to behavior, are key factors in determining personal health behaviors (13). Therefore, health awareness among the older adults can be viewed as a perception of health threats, while the acquisition, understanding, and application of health information influence the perception of the benefits and barriers to health behaviors, as well as self-efficacy in managing one’s health. These factors work together to shape the health awareness of the older adults, ultimately affecting their health behaviors and outcomes.

Rowlands et al. studied the barriers to obtaining and applying health information and pointed out that although it is becoming easier to obtain health information, understanding and effectively applying this information remains a key challenge to achieving good health outcomes (14). It can be seen that the health information processing process has an important impact on the formation of health awareness.

Specifically, first, by obtaining health information, the older adults increase their awareness of potential health problems and their understanding of health behaviors. This information acquisition improves their perception of health threats (corresponding to perceived susceptibility in HBM) and their cognition of health behaviors (corresponding to perceived benefits), thereby improving health awareness. Jones et al. used the health belief model to explore parents’ attitudes toward childhood vaccination (15). The study showed that the effective transmission and application of health information can enhance individuals’ self-efficacy and positive cognition of health behaviors, thereby improving the implementation rate of health behaviors (16).

Secondly, the older adults understand and screen the information they obtain, which helps them judge which health information is credible and useful. This process is closely related to the perceived severity and perceived barriers in HBM. Champion et al. explored how the health belief model affects the participation rate of breast cancer screening in women and found that perceived susceptibility and perceived barriers are important factors in predicting health behavior (17), and that these barriers can be significantly reduced and screening participation rates can be increased through health education and information transmission. The process of understanding information can enhance the confidence and ability of the older adults to adopt healthy behaviors and further enhance their health awareness.

Then, the process of applying health information is the key step for the older adults to transform information into actual healthy behaviors. Through the actual application of information, they can see the actual effects of healthy behaviors, which is directly related to the self-efficacy in HBM and further enhances health awareness.

Finally, in HBM, enhanced health awareness will prompt individuals to adopt more positive health behaviors and ultimately improve health outcomes. Through enhanced health awareness, the older adults pay more attention to their own health and take corresponding measures to maintain and improve their health. Hagger et al.’s research shows that the perceived benefits and self-efficacy in the health belief model (18) are closely related to the autonomous motivation in the self-determination theory. By enhancing individuals’ sense of control and autonomy over health behaviors, it can more effectively promote changes in health behaviors (19).

From the current literature analysis, existing research mainly focuses on how health awareness drives the acquisition, understanding and application of information, while this study starts from the other end of the causal relationship and explores how the information processing process shapes health awareness. This reverse exploration provides a new perspective for understanding the formation of health awareness, revealing that health awareness is not innate, but can be cultivated and enhanced through information acquisition, understanding and application. Therefore, the core issue of this study is to explore how the acquisition, understanding and application of health information affect the health awareness of the older adults, and at the same time, health awareness plays a mediating role between health information behavior and health outcomes. This study not only helps to enrich the theoretical connotation of the health belief model, but also provides a new practical guidance direction for the health management of the older adults.

### 1.2 Formulation of research hypotheses

Based on an analysis of relevant studies, this research proposes a theoretical model aimed at exploring the relationships between the acquisition, understanding, and application of health information and the health awareness and health outcomes of the older adults. The model is grounded in the Health Belief Model (HBM), which has been widely used to explain the determinants of individual health behaviors (20). In existing research, health literacy is considered a key factor influencing health behaviors and outcomes. Although these studies have examined the role of acquiring, understanding, and applying health information in health behaviors, they primarily focus on overall health literacy and do not sufficiently analyze how the information processing process specifically affects the health awareness of the older adults and how it indirectly influences health outcomes through health awareness. Based on the HBM theory, this study hypothesizes that the acquisition, understanding, and application of health information can directly enhance the health awareness of the older adults, and more importantly, that health awareness serves as a mediating variable, indirectly improving health outcomes by influencing the health behaviors of the older adults. Based on this, the study proposes the following specific hypotheses:

*H1*: The acquisition of health information has a significant positive impact on the health awareness of the older adults.

*H2*: The understanding of health information has a significant positive impact on the health awareness of the older adults.

*H3*: The application of health information has a significant positive impact on the health awareness of the older adults.

*H4*: Health awareness has a significant positive impact on the health outcomes of the older adults.

*H5*: The acquisition of health information indirectly affects health outcomes by enhancing health awareness.

*H6*: The understanding of health information indirectly affects health outcomes by enhancing health awareness.

*H7*: The application of health information indirectly affects health outcomes by enhancing health awareness.

The specific explanations of the research hypotheses are as follows:

H1: The acquisition of health information has a significant positive impact on the health awareness of the older adults. When older adults individuals actively acquire health information, such as by searching for information online (Question 10) or obtaining information through social media (Question 12), they become more attentive to their health, thereby enhancing their health awareness (e.g., Questions 1–8). The Health Belief Model (HBM) emphasizes that personal health behaviors are determined by perceived threats (21). The process of acquiring information helps the older adults recognize the necessity of health management, thereby influencing their behavioral intentions and actions.

H2: The understanding of health information has a significant positive impact on the health awareness of the older adults. When older adults individuals can effectively understand and evaluate the health information they acquire (e.g., filtering and processing information, Question 17; assessing the credibility of information, Question 19), they are more likely to recognize the importance of this information to their health (22). This understanding process helps deepen their awareness of health issues, thereby enhancing their health awareness.

H3: The application of health information has a significant positive impact on the health awareness of the older adults. When older adults individuals apply health information to their daily lives (e.g., using the information to correct unhealthy habits, Question 26; or developing a personal health plan, Question 28), they become more aware of the practical benefits of health management (23). According to the HBM theory, when individuals successfully apply health information and observe positive outcomes, their perceived benefits of health behaviors are enhanced, and their perceived self-efficacy increases, meaning their confidence in successfully executing health behaviors is boosted. This positive feedback from successful application not only reinforces their health cognition but also further enhances their health awareness, as they increasingly recognize the actual value and effectiveness of adopting these health behaviors.

H4: Health awareness has a significant positive impact on the health outcomes of the older adults. Older adult individuals with higher health awareness are more likely to engage in positive health behaviors, such as adjusting their lifestyle (Question 33) or effectively managing their health conditions (Question 36) (24). According to the HBM theory, an individual’s health behavior is influenced by their perception of health threats, perceived benefits of health behaviors, self-efficacy, and perceived barriers to behavior. Higher health awareness typically means that older adult individuals have a stronger perception of potential health threats and a clearer understanding of the benefits that health behaviors can bring. This enhanced awareness encourages them to more actively utilize the information they acquire, understand, and apply, thereby more effectively managing their health conditions and ultimately improving their overall health outcomes.

H5: The acquisition of health information indirectly affects health outcomes by enhancing health awareness. Older adults individuals who actively acquire health information (e.g., Question 10, searching for health information online) enhance their health awareness (e.g., Question 1, actively seeking health information) (25). This heightened awareness further influences their health outcomes (e.g., Question 33, adjusting lifestyle habits based on the acquired information). This suggests that the acquisition of health information not only directly impacts awareness but also indirectly affects health outcomes through enhanced awareness.

H6: The understanding of health information indirectly affects health outcomes by enhancing health awareness. When older adults individuals can effectively understand the health information they acquire (e.g., Question 18, understanding the specific meaning of information), their health awareness is enhanced (e.g., Question 3, willingness to obtain health information through the media). This increase in awareness makes them more attentive to their health, thereby improving health outcomes (e.g., Question 35, improving health conditions by understanding the information) (26).

H7: The application of health information indirectly affects health outcomes by enhancing health awareness. When older adults individuals successfully apply health information in their lives (e.g., Question 27, sharing health information with family and friends), their health awareness is further enhanced (e.g., Question 5, willingness to learn and improve health awareness) (27). The positive experiences and feedback from this practice lead older adult individuals to place greater importance on health information, thereby forming higher health awareness on both cognitive and behavioral levels. This enhanced awareness contributes to more effective health management (e.g., Question 36, managing health by applying information) (28), ultimately improving health outcomes. The HBM theory explains that the application of health information not only helps older adult individuals recognize the benefits of health behaviors but also increases their self-efficacy and perception of health threats, reducing perceived barriers to behavior, thus indirectly enhancing health awareness and ultimately improving health outcomes.

**Figure 2:**
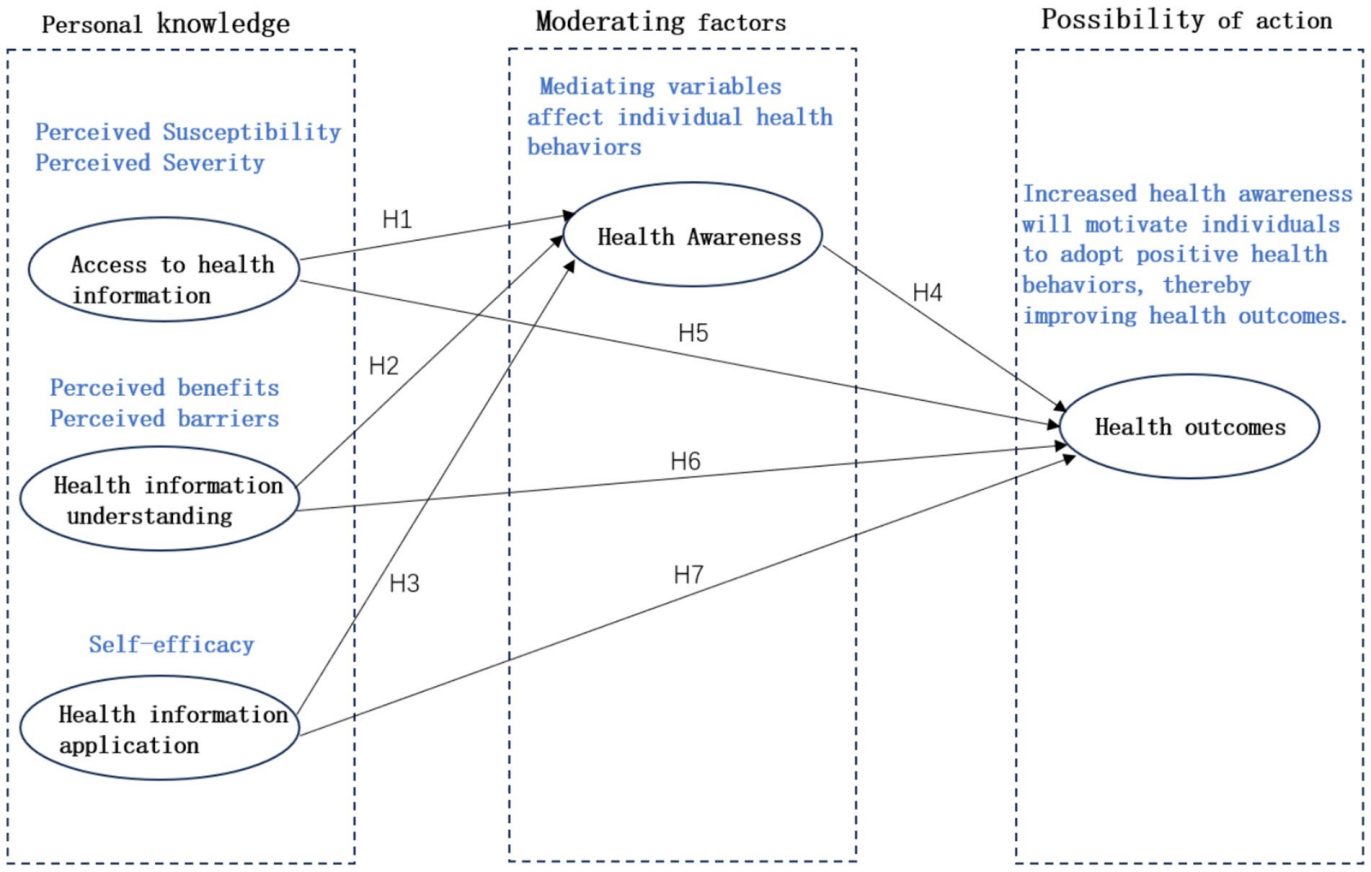
Research hypothesis model.

Based on the above hypothesized relationships, this study integrates the HBM theory to propose a theoretical model of research hypotheses, as shown in Figure 2.

Figure 2 shows the relationship and interaction mechanism between the acquisition of health information, understanding of health information, application of health information, health awareness and health outcomes. The theoretical model shows a multi-path causal relationship, describing how to improve health outcomes by improving the acquisition, understanding and application of health information by the older adults, thereby enhancing health awareness. In the future, these hypotheses will be verified through structural equation modeling (SEM), and the interaction mechanism between these variables will be deeply understood to provide a theoretical basis for the formulation of health management strategies for the older adults.

## 2 Research methodology

### 2.1 Questionnaire design

This study employs a rigorous empirical research approach for analysis. Previous studies on health information literacy among the older adults have commonly utilized scales such as the eHealth Literacy Scale (eHEALS) and the Digital Health Literacy Scale (DHLI) (29). The eHealth Literacy Scale is used to assess an individual’s comprehensive knowledge, comfort, and perceived skills in finding, evaluating, and applying electronic health information to solve health problems. The Digital Health Literacy Scale, developed by Van der Vaart and Drossaert in 2017, evaluates areas including operational skills, information search capabilities, evaluation skills, and understanding, and can be used to assess the older adult’s utilization of electronic health resources (30). However, neither of these scales addresses the dimension of health awareness. Therefore, to better align with the specific objectives and research hypotheses of this study, a new questionnaire tool was developed, drawing on the eHealth Literacy Scale and the Digital Health Literacy Scale.

Firstly, existing standardized scales are often tailored to specific contexts and may not fully capture the unique challenges and characteristics faced by the older adults in the process of acquiring, understanding, and applying health information. Secondly, the hypotheses proposed in this study involve three distinct dimensions of health information processing—acquisition, understanding, and application—and their direct and indirect effects on health awareness and outcomes. While these dimensions partially overlap with the content measured by traditional scales, they differ in focus and specific measurement items. Using existing scales directly would not fully cover the measurement content required by this study. Finally, this study, based on the Health Belief Model (HBM), constructs a new theoretical model aimed at exploring the causal relationships between health information literacy (acquisition, understanding, and application) and health outcomes among the older adults. To ensure the compatibility of the measurement tool with the theoretical model, it is necessary to develop a scale that reflects the constructs within the model, facilitating verification and analysis through Structural Equation Modeling (SEM). Therefore, to more accurately measure the health information literacy of the older adults and its impact on health awareness and outcomes, it was necessary to construct a questionnaire tailored to the specific needs of this study.

To ensure the scientific validity and reliability of the scale, the Delphi method was employed to determine the content and structure of the scale (31). Seven experts with extensive research experience in health information literacy, older adult health management, and psychology were invited. The experts included three health education scholars, two geriatric medicine specialists, and two social psychology experts, ensuring a multidisciplinary integration and comprehensiveness of the scale content. After the initial design of the scale, the first round of the questionnaire was distributed to these experts, seeking their opinions on the structure, question design, and content validity of the scale. The experts were asked to evaluate the relevance, clarity, and applicability of each question and provide specific suggestions for revisions. The collected expert opinions were then summarized and analyzed to assess the degree of consensus among the experts on each question. For questions with significant expert disagreement, the underlying reasons were recorded and analyzed. The questionnaire developed for this study consists of 39 questions divided into two parts. The first part includes three questions regarding the respondent’s basic personal information, such as gender, age, and education level. The second part is the Health Information Literacy Scale for the older adults, comprising 36 questions. The results indicated that the experts generally agreed with the overall framework of the initially designed scale, but suggested adjustments to the wording and order of certain questions.

In the section on the Health Information Literacy Scale for the older adults, in line with the Health Belief Model theory, 16 questions were set across four aspects: health information awareness, health information acquisition, health information understanding, and health information application (Questions 4–7, 12–15, 20–23, 28–31). Additionally, to measure the impact of learning and website design on the health information literacy of the older adults, eight questions were set to assess the older adult’s learning of health information (two questions for each aspect: awareness, acquisition, understanding, and application), and another eight questions were set to measure the older adult’s responses to website design (two questions for each aspect: awareness, acquisition, understanding, and application). The Health Information Outcomes Scale for the older adults includes four questions (Questions 36–39) to measure the outcomes of health information application. All 36 measurement questions in the questionnaire use a five-point Likert scale, with options ranging from “strongly disagree,” “disagree,” “neutral,” “agree,” to “strongly agree,” and are scored from 1 to 5, respectively. The questionnaire, based on existing mature scales, comprehensively examines the health literacy of the older adults across the four dimensions of health information literacy and health literacy outcomes (32), while also incorporating the elements of learning and design. The questionnaire comprehensively considers the entire process of health information literacy among the older adults, offering robust coverage. It integrates individual subjectivity with external environmental factors, emphasizing the interaction and mutual influence of personal health awareness with personal, material, and environmental factors.

### 2.2 Data collection and processing

This survey utilized a convenience sampling method. To better ensure the validity of the questionnaire, two pilot surveys were conducted. The first pilot survey was carried out on a small scale, resulting in 53 collected questionnaires. The second pilot survey was conducted among older adults individuals in communities around Guangzhou, with 89 questionnaires collected. Based on the feedback from these pilot surveys, the questionnaire was refined to produce the final version. In the formal survey, the target population was set as older adults individuals (aged 60 and above). A total of 400 questionnaires were distributed, with 360 returned. After screening the questionnaires based on the response time (greater than 60 s), 341 valid questionnaires were obtained, resulting in a recovery rate of 90% and an effective rate of 85.25%, meeting the designed expectations. This study was approved by the Ethics Committee of Guangzhou Huashang College, and informed consent was obtained from all participants.

The data from the 341 valid questionnaires were imported into SPSS software for reliability and validity analysis of the 36 questions (as shown in Table 1). The analysis results indicate that the Cronbach’s *α* for the reliability test is 0.987, demonstrating that the survey data have very high reliability and good internal consistency. The KMO statistic for the validity test is 0.985, which is greater than 0.8, indicating that the survey data are highly suitable for factor extraction. The Bartlett’s test of sphericity yielded a value of 14055.463 (df = 630, Sig = 0.000), reaching a significant level, which indicates that the questionnaire items have good measurement validity and are appropriate for factor analysis and structural equation modeling.

**Table 1:** Reliability and validity test results of health information literacy and results for the older adults.

Reliability test		Validity test		
Cronbach’s Alpha	0.987	The sampling adequacy measure, Kaiser-Meyer-Olkin (KMO)		0.985
		Bartlett’s test of sphericity	Approximate Chi-Square	14055.463
Number of items	36		df	630
			Sig.	0.000

### 2.3 Preliminary analysis of the questionnaire

#### 2.3.1 Analysis of respondents’ basic information

An analysis of the basic information of the respondents revealed that out of the 341 valid questionnaires, 153 respondents were male, and 188 were female, with females slightly outnumbering males and the gender ratio close to 1:1, which is consistent with the current structure of the older adults population in China. There were 105 respondents aged 60–64, accounting for 30.8%; 92 respondents aged 65–69, accounting for 27%; 64 respondents aged 70–74, accounting for 18.8%; 53 respondents aged 75–79, accounting for 15.5%; and 27 respondents aged 80 and above, accounting for 79%, closely matching the current age structure of the older adults population in China. Among the respondents, 42 had no formal education, 93 had completed primary education, 75 had completed junior high school, 82 had completed high school, and 49 had an associate degree or higher, reflecting a distribution similar to the current educational structure of the older adults population in China.

#### 2.3.2 Descriptive analysis of health information literacy among the older adults

The values for the older adult’s health information awareness, acquisition, understanding, application, and outcomes were calculated by summing the respective scores of the four measurement questions in each of these five aspects. Descriptive analysis was then conducted, resulting in the findings shown. From Table 2, it can be observed that all five variables exhibit a certain degree of left-skewness. Additionally, awareness, application, and outcomes show a certain degree of kurtosis, while acquisition and understanding display mild platykurtic tendencies.

**Table 2:** Descriptive analysis of health information literacy of the older adults.

Variable	Minimum	Maximum	Mean	Standard Deviation	Skewness	Kurtosis
Awareness	4.00	20.00	16.1848	4.20691	−1.438	1.130
Acquisition	4.00	20.00	15.3402	4.39670	−0.956	−0.215
Understanding	4.00	20.00	15.2698	4.37680	−0.959	−0.097
Application	4.00	20.00	15.7478	4.13675	−1.249	0.684
Results	5.00	20.00	16.0147	4.03766	−1.315	0.855

### 2.4 Structural equation modeling validation

Structural Equation Modeling (SEM) was used to validate the hypothesized model. By comparing the theoretical model with the empirical results, it is evident that most of the hypotheses were supported, with the impact of health information application on health awareness and health outcomes being significantly higher than other factors. Several goodness-of-fit indices were used to assess the overall fit of the model, including Chi-square (χ^2^), Degrees of Freedom (DF), Root Mean Square Error of Approximation (RMSEA), Normed Fit Index (NFI), and Comparative Fit Index (CFI), among others (33). These indices indicate that the model has a good fit, suggesting that the SEM can reasonably explain the relationships between health information behaviors, health awareness, and health outcomes. The specifics are as follows.

#### 2.4.1 Direct paths

Health Information Acquisition → Health Awareness (H1)

Health Information Understanding → Health Awareness (H2)

Health Information Application → Health Awareness (H3)

Health Awareness → Health Outcomes (H4).

**Figure 3:**
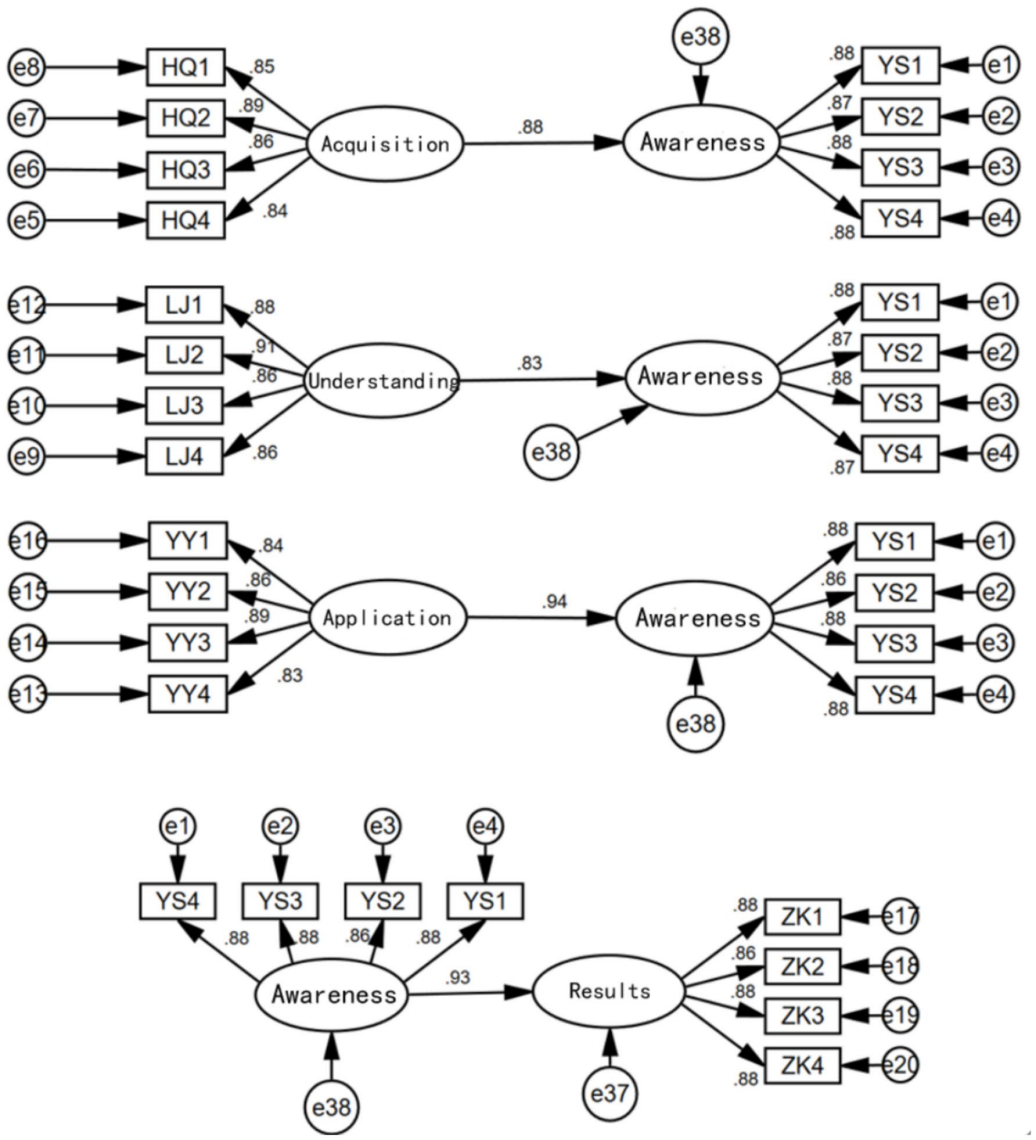
Direct path relationship diagram.

Based on the analysis, separate models were constructed to examine the effects of health information acquisition on health awareness, health information understanding on health awareness, health information application on health awareness, and health awareness on health outcomes. The results are shown in Figure 3. From the results, it can be seen that health information acquisition has a significant positive impact on health awareness (0.88, supporting Hypothesis 1), health information understanding has a significant positive impact on health awareness (0.83, supporting Hypothesis 2), and health information application has a significant positive impact on health awareness (0.94, supporting Hypothesis 3). The order of influence is as follows: Health Information Application > Health Information Acquisition > Health Information Understanding. Health awareness also has a significant positive impact on health outcomes (0.93, supporting Hypothesis 4).

The strong influence of health information application on health awareness may be due to the direct improvement in physical health conditions resulting from the application of health information, which makes the older adults more aware of the importance of health information, thereby greatly enhancing their health awareness. Understanding health information is a long-term process, with significant differences in how individuals from different backgrounds interpret information. Additionally, there are considerable disparities in the cultural and educational levels between urban and rural older adult populations (34), which may contribute to the relatively smaller impact of health information understanding on health awareness among the three factors.

**Figure 4:**
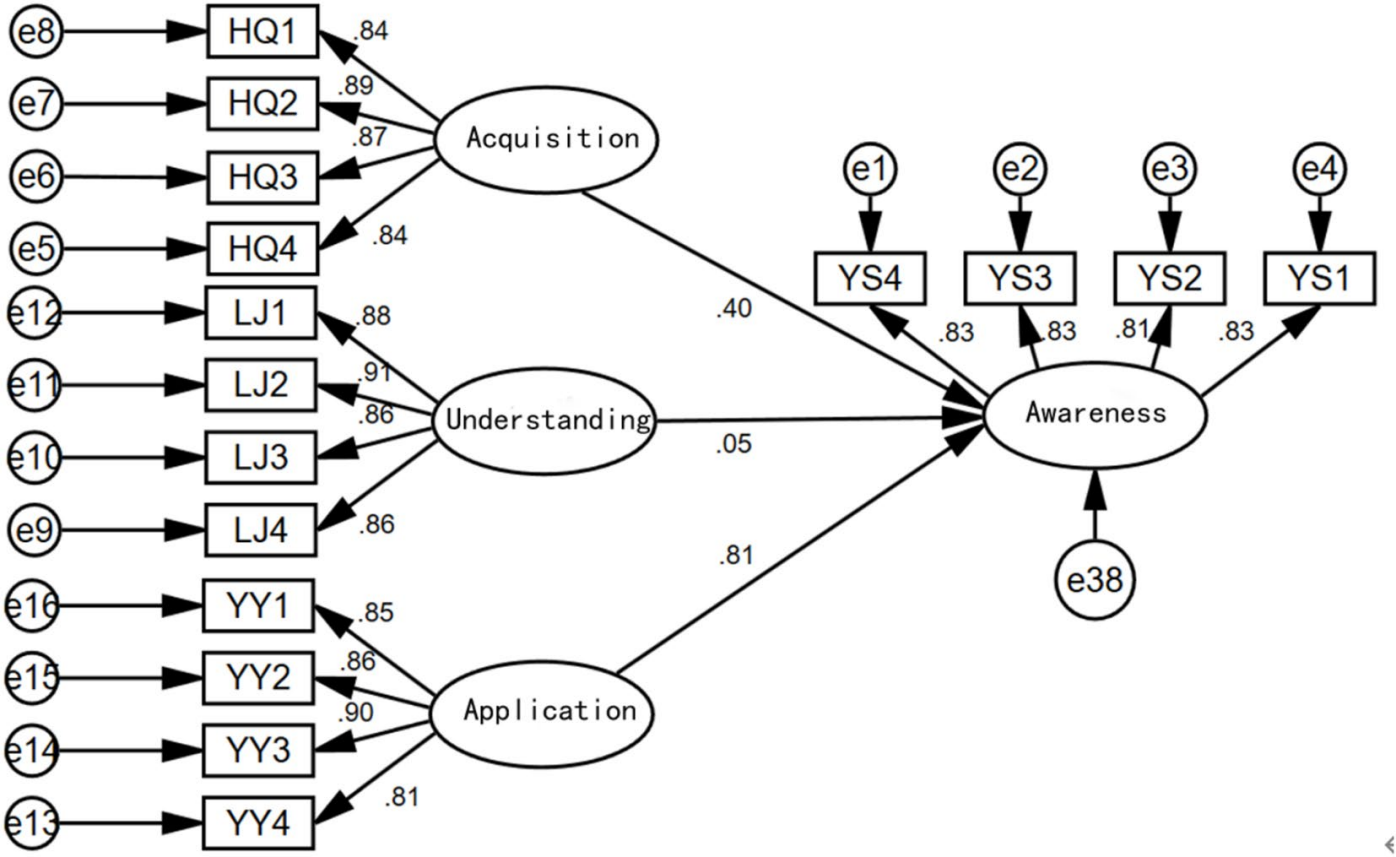
Influence diagram considering all three factors simultaneously.

After separately considering the effects of health information acquisition, understanding, and application on health awareness, we constructed a structural equation model that includes all three factors simultaneously, as shown in Figure 4.

From the results shown above, when considering health information acquisition, understanding, and application simultaneously, health information acquisition and application both have significant positive impacts on health awareness. However, the impact of health information understanding on health awareness is not significant (with a coefficient of 0.05 and a *p*-value of 0.188 > 0.05, indicating insignificance). The direct influence order among the three factors remains the same as when considered separately: Health Information Application > Health Information Acquisition > Health Information Understanding.

#### 2.4.2 Indirect paths

Health Information Acquisition → Health Awareness → Health Outcomes (H5)

Health Information Understanding → Health Awareness → Health Outcomes (H6)

Health Information Application → Health Awareness → Health Outcomes (H7).

To further consider the impact of health information acquisition, understanding, and application on the health status of the older adults, and to explore the mediating role of health awareness, the following mediation effect models were established for exploration.

**Figure 5:**
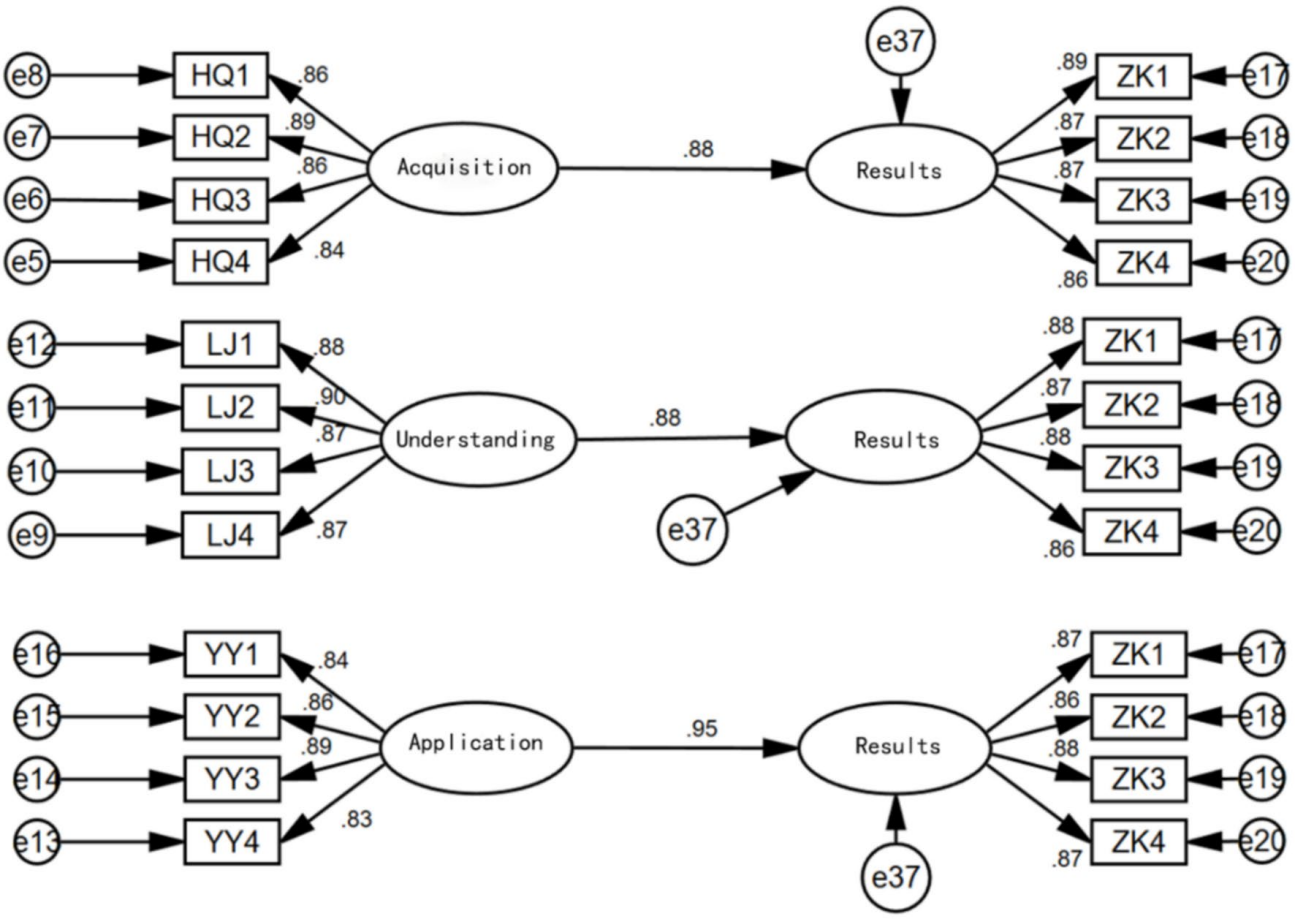
The effects of health information acquisition, understanding, and application on health outcomes.

First, separate models were constructed to examine the effects of health information acquisition on health status, health information understanding on health status, and health information application on health status, as shown in Figure 5. The results indicate that health information acquisition, understanding, and application all have significant positive impacts on the health status of the older adults, with standardized coefficients of 0.88, 0.88, and 0.95, respectively.

**Figure 6:**
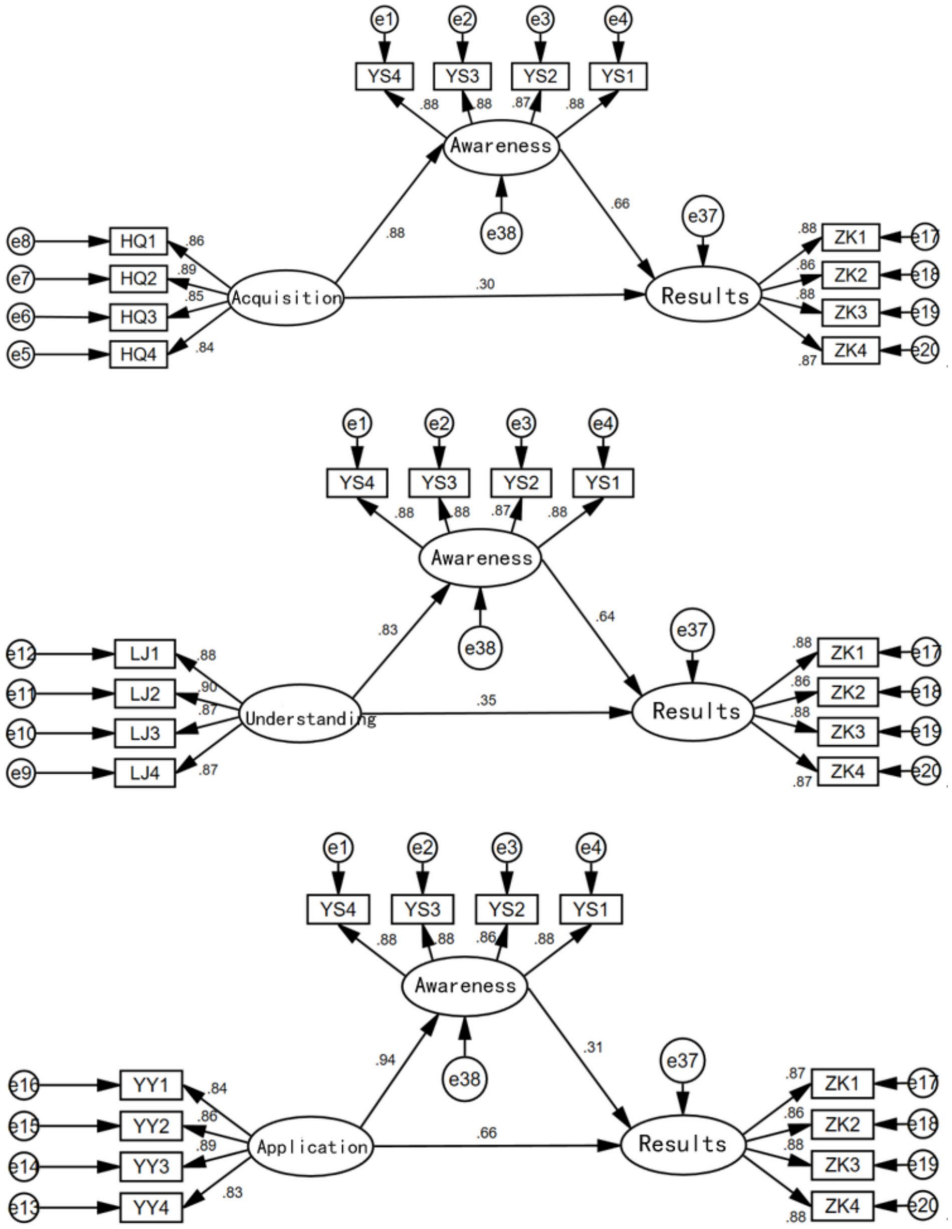
Health awareness as a mediating variable.

In each of the above models, health awareness was added as a mediating variable, and the results are shown in Figure 6.

From the results in Figure 6, it is evident that health awareness has a significant mediating effect in the relationships between health information acquisition and health outcomes, health information understanding and health outcomes, and health information application and health outcomes (supporting Hypotheses 5, 6, and 7). The direct and mediating effects of each model are shown in Table 3. It can be seen that the mediating effect of health awareness is greatest in the model of health information acquisition on health outcomes (0.58), followed by the model of health information understanding on health outcomes (0.53), and smallest in the model of health information application on health outcomes (0.29).

**Table 3:** The mediating effect of health awareness in each model.

Model	Direct effect	Mediating effect of health awareness
Get → Results	0.30	0.88 × 0.66 = 0.58
Understand → Results	0.35	0.83 × 0.64 = 0.53
Apply → Results	0.66	0.94 × 0.31 = 0.29

**Figure 7:**
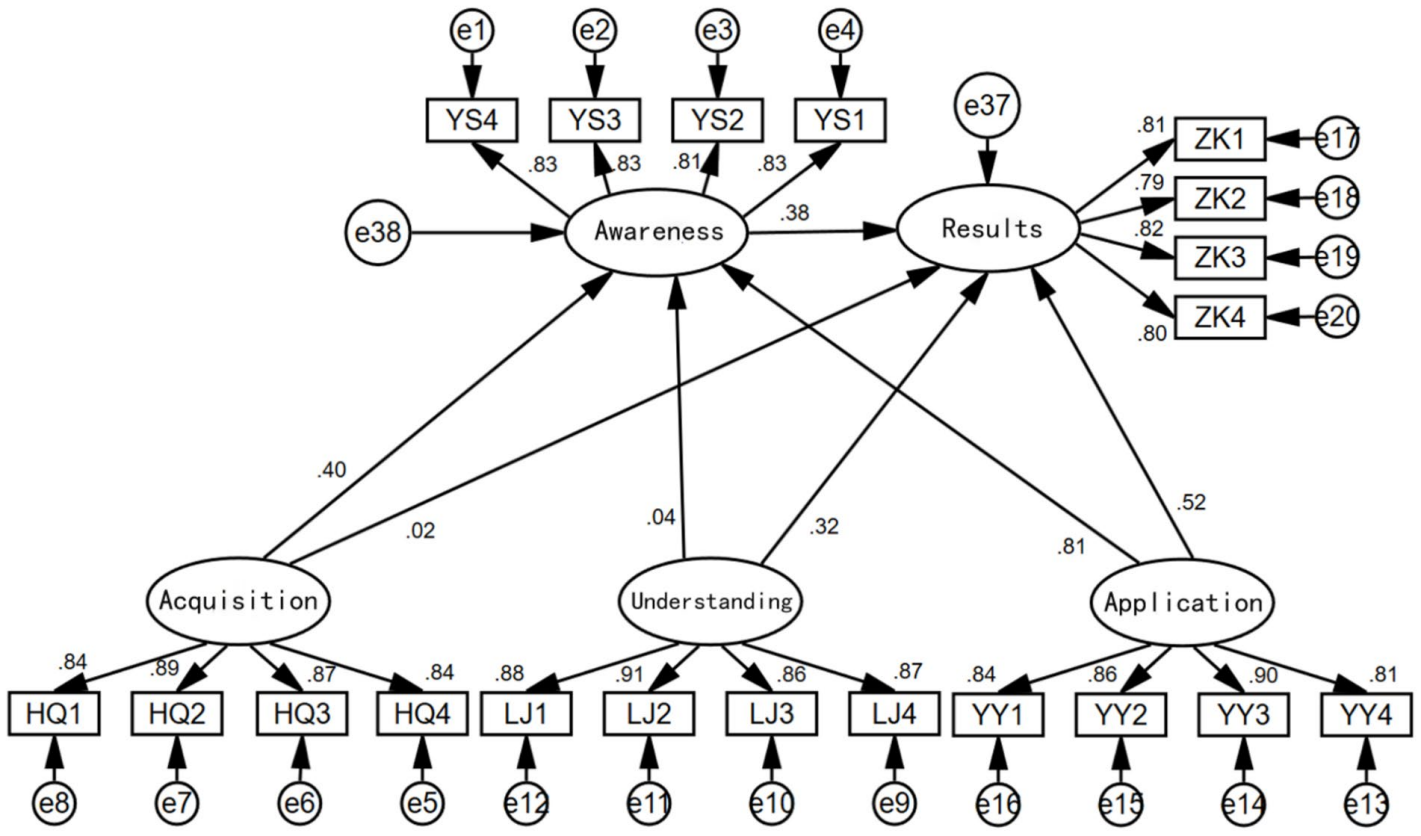
Model testing results.

When considering the simultaneous effects of health information acquisition, understanding, and application on health outcomes, a model was constructed with awareness as the mediating variable. From the results in Figure 7, it is evident that when all three factors are considered simultaneously, the direct effect of health information application on health outcomes is the largest (0.52), followed by the direct effect of health information understanding on health outcomes (0.32). The direct effect of health information acquisition on health outcomes is not significant (0.02 with a *p*-value of 0.649 > 0.05, indicating insignificance).

The mediating effect of health awareness in the model of health information application on health outcomes is significant (0.81 × 0.38 = 0.31), ranking first; the mediating effect of health awareness in the model of health information acquisition on health outcomes is also significant (0.40 × 0.38 = 0.152), ranking second; however, the mediating effect of health awareness in the model of health information understanding on health outcomes is not significant. Initially, the “sequential test” was used to examine the mediating effect, with a p-value of 0.04 corresponding to 0.215 > 0.05, indicating insignificance, preventing awareness from forming a mediating effect between understanding and outcomes. Further, the “Sobel test” was applied (35), and the calculation yielded the following results:

$S_{\mathrm{ab}}=\sqrt{{\hat{a}}^{2}s_{b}^{2}+{\hat{b}}^{2}s_{a}^{2}}=0.009963$ , $z=\frac{\hat{a}\hat{b}}{S_{\mathrm{ab}}}=1.199$ < $z_{\alpha /2}=1.96.$

Therefore, the Sobel test failed and the mediation effect was not significant.

## 3 Results

This study used Structural Equation Modeling (SEM) to analyze the impact of health information acquisition, understanding, and application on the health awareness of the older adults and to further explore the influence of health awareness on health outcomes. The results are as follows:

### 3.1 Direct effects

*H1*: Health information acquisition positively impacts health awareness, with a path coefficient of 0.88.

*H2*: Health information understanding positively impacts health awareness, though to a lesser extent, with a path coefficient of 0.83.

*H3*: Health information application has the strongest positive impact on health awareness, with a path coefficient of 0.94.

*H4*: Health awareness significantly improves health outcomes, with a path coefficient of 0.93.

### 3.2 Mediation effects

To assess the mediation effects, the study tested the role of health awareness as a mediator between the different health information factors (acquisition, understanding, and application) and health outcomes. The single-factor mediation model revealed that health awareness significantly mediates the relationship between health information behaviors and health outcomes, except in the model where health information understanding influenced health outcomes. Specifically, health awareness was shown to have a significant positive mediating effect in all but the understanding model.

In the multi-factor mediation effect model, health awareness was found to have a significant mediating effect when health information acquisition and application influenced health outcomes, while it was not significant in the model where health information understanding influenced health outcomes. This highlights that health awareness plays a crucial role in bridging the gap between health information behaviors and health outcomes for acquisition and application, but not for understanding.

### 3.3 Indirect effects

*H5*: Health information acquisition indirectly influences health outcomes through health awareness, with an indirect effect of 0.58.

*H6*: Health information understanding has a moderate indirect effect on health outcomes via health awareness, with an indirect effect of 0.53.

*H7*: Health information application has the largest indirect effect on health outcomes through health awareness, with an indirect effect of 0.29.

When considering all three factors simultaneously, health information acquisition and application significantly influenced health awareness, while understanding did not (path coefficient = 0.05, *p* = 0.188). The indirect effects confirm that health awareness mediates the relationship between health information behaviors and health outcomes, with the strongest mediation from acquisition (0.58), followed by understanding (0.53), and application (0.29).

In summary, the study shows that the application of health information has the most significant effect on health awareness, followed by the acquisition of health information. Understanding plays a comparatively lesser role in directly influencing health awareness. Furthermore, health awareness acts as a significant mediator in the relationship between health information behaviors and health outcomes, with the greatest mediation effect observed for acquisition.

## 4 Discussion

This study found that health information acquisition, understanding, and application all significantly influence the health awareness of the older adults, and through health awareness, they also impact their health outcomes. Acquiring health information is the first step for the older adults to enhance their health awareness. The more diverse the channels and the higher the quality of the information they obtain, the deeper their understanding of health management (36). In an information-rich society, particularly for the older adults, the ability to easily access reliable health information is crucial. Among these factors, the application of health information has the most significant impact on improving health awareness. This is because when the older adults see the direct benefits of health behaviors in practice, it boosts their confidence, leading them to believe that they can improve their health by applying the information. This increase in confidence, in turn, further enhances their health information awareness.

This finding suggests that in the process of acquiring health information, the older adults especially need to see tangible health improvements, which can motivate them to participate more actively in health management. The study reveals that the way health information is applied is the most critical factor in enhancing health awareness, thereby helping to optimize the methods of health information delivery. This provides empirical evidence for public health policymakers, facilitating the design of more targeted intervention measures. In terms of health education for the older adults, there should be a greater focus on practical guidance and application support. These findings could encourage policymakers to adjust resource allocation, placing more emphasis on areas that can maximize the improvement of health awareness.

Within the framework of the Structural Equation Model, the impact of health information acquisition, understanding, and application on health outcomes is not only direct but also involves indirect effects through the mediating variable of health awareness. Specifically, information acquisition exposes the older adults to necessary health knowledge, understanding allows them to better digest and internalize this knowledge, and application converts the knowledge into actual behavior. The key finding here is that health awareness acts as a crucial mediating variable, linking these information processing processes to health outcomes. Model analysis indicates that the enhancement of health awareness significantly strengthens the impact of information acquisition, understanding, and application on health outcomes, confirming the existence and importance of this indirect effect. Although health information acquisition and understanding also have significant effects on health awareness, their influence is relatively smaller. This may be due to the cognitive decline associated with aging and the limited channels through which the older adults can acquire health information. The older adults often rely on traditional media or a limited social network for information, and the way these channels deliver information may not fully stimulate their health awareness. Moreover, the difficulty in understanding information also hinders the older adults from internalizing it into action, which is related to the complexity of health information.

The results of this study are consistent with the Health Belief Model (HBM) theory, supporting the view that health awareness serves as a mediator in health behaviors and outcomes. According to the HBM theory, an individual’s health behavior is driven by their perceived threat of health issues, perceived benefits and barriers to health behaviors, and self-efficacy (37). This study shows that through the acquisition, understanding, and application of health information, the older adults gradually enhance their perception of health threats and confidence in health behaviors, thereby improving their health awareness. This is consistent with the HBM concepts of perceived susceptibility, perceived benefits, and self-efficacy.

Unlike some previous studies, this study highlights the central role of health information application in enhancing health awareness. While other studies have focused more on the role of information acquisition and understanding in health management, this study found that the direct practical effects of information application are more significant. This difference may stem from the fact that the study population consists of older adult individuals who tend to increase their trust and reliance on information after seeing actual results (38). Through this research perspective, it is evident that health awareness is actually a dynamic process, strongly influenced by external information stimuli. This suggests that by improving the acquisition, understanding, and application of health information, significant improvements in health awareness can be achieved, thereby influencing health behaviors and outcomes. This provides new strategic directions for public health interventions, suggesting that optimizing information delivery and educational methods can enhance the health awareness of the older adults.

While this study provides valuable insights into the impact of health information behavior on the health awareness and outcomes of the older adults, it does have certain limitations. One limitation is that the representativeness of the sample could not be fully validated due to the lack of baseline data on the target population. Although the sample size of 360, determined by feasibility constraints, is generally sufficient for detecting moderate effects, the study’s sample may not fully capture the diversity of the broader older adult population. However, the sample demographics (e.g., gender, age, education) are consistent with available data on the target population, and the study achieved a high response rate (90%). We believe that this limitation does not significantly affect the validity of the study’s overall conclusions. Future research could benefit from exploring alternative sampling methods to further strengthen the generalizability and external validity of the findings. Nevertheless, the conclusions of this study remain robust and applicable to the context under investigation.

## 5 Conclusion and recommendations

### 5.1 Conclusion

This study, through empirical analysis, revealed the critical role of health information acquisition, understanding, and application in enhancing the health awareness of the older adults. It further explored the mediating role of health awareness in the relationship between older adult health information behaviors and health outcomes. The results clearly indicate that health information acquisition, understanding, and application not only directly enhance the health awareness of the older adults but also significantly connect and strengthen the relationship between health information behaviors and health outcomes through the mediating variable of health awareness. Specifically, health awareness plays a dual role in this process: firstly, it acts as an internal motivation, driving the older adults to engage more actively in health management. Secondly, it directly influences health outcomes, with higher health awareness being associated with a greater likelihood of the older adults taking proactive health actions, such as regular check-ups, following medical advice, and adjusting unhealthy lifestyles. This proactivity is closely related to health outcomes, as the study shows that individuals with higher health awareness are more willing to participate in health promotion activities and follow health guidelines, thereby significantly improving their overall health status. This finding provides important theoretical support and practical guidance for advancing the “Healthy Aging” strategy, emphasizing the importance of health awareness as a mediating variable. Based on these findings, enhancing health awareness should become a key priority in public health policy.

### 5.2 Recommendations

Based on the conclusions of this study, five key strategies are proposed to effectively enhance the health awareness of the older adults and improve their health outcomes. These strategies aim to comprehensively utilize modern technology and social resources to address the health challenges posed by an aging society in the era of artificial intelligence through systematic interventions.

#### 5.2.1 Promote digital health platforms to enhance the health information acquisition and application abilities of the older adults

The study shows that acquiring and applying health information plays a crucial role in enhancing the health awareness of the older adults. To fully leverage the role of digital health platforms in this process, the government should promote the development and dissemination of health applications and online platforms specifically designed for the older adults. These platforms should simplify interfaces and operational processes, fully considering the changes in the older adult’s vision, hearing, and cognitive abilities. This could include using larger fonts, simplified icons, intuitive navigation, and providing voice assistance features to enable the older adults to easily access, understand, and manage health information. Additionally, platform design should incorporate personalized features, such as customized health reminders, personalized health advice, and easy-to-use health record systems, to enhance the older adult’s ability to apply health information and, in turn, improve their health awareness. The platform should also include interactive elements, such as online consultations, telemedicine, and health communities, allowing the older adults not only to obtain information but also to interact with healthcare professionals and peers, thereby increasing their motivation for health behaviors.

The government should also collaborate closely with community healthcare institutions and eldercare service providers to promote the use of these platforms within communities and provide ongoing technical support and services to the older adults. This includes establishing technical help centers within communities, organizing digital health training classes, and arranging for professionals to offer in-home guidance to ensure that the older adults can smoothly use these platforms and continuously benefit from the convenience of digital health tools. Through these initiatives, the older adults can better acquire and apply health information, effectively enhancing their health awareness and overall health management capabilities.

#### 5.2.2 Strengthen community health education and interaction to enhance the health awareness and behavioral motivation of the older adults

The community is an important setting for the daily lives of the older adults, and conducting community health education and interactive activities is a key strategy for enhancing their health awareness. The government should support and organize regular community health lectures, free clinics, and health knowledge competitions to stimulate the older adult’s interest in and attention to health information. Additionally, community health support groups should be established to encourage the exchange of health information and experience sharing among the older adults, creating a positive environment for health behaviors. Successful cases in this area include Singapore’s “Health Promotion and Advocacy Program” and Hong Kong’s “Older adults Health Program.” Singapore’s program involves appointing “Health Advocates,” who are either health enthusiasts within the community or trained volunteers (39), to lead the older adults in health activities such as fitness exercises and walking clubs, effectively increasing their engagement in health management. Hong Kong’s “Older adults Health Program,” led by the Hong Kong Department of Health, offers comprehensive health services for the older adults, including health check-ups, health education, and chronic disease management through “Older adults Health Centers” in community health centers. Such community-based health education not only enhances the health awareness of the older adults but also significantly boosts their motivation for health behaviors, promoting the sustainability of health management.

#### 5.2.3 Utilize multimedia channels to improve the accessibility of health information dissemination

The effectiveness of health information dissemination directly affects the health awareness and behaviors of the older adults (40). Based on the findings of this study, information acquisition is an important pathway for enhancing health awareness. The government should fully utilize multimedia channels such as television, radio, and social media to widely promote scientific and credible health information. For the older adults population, health-themed programs and short videos should be developed and promoted to ensure that the content is easy to understand and practically useful. Additionally, the government should compile and distribute health booklets that cover common health issues and management methods for the older adults, ensuring the accessibility of the information. Through these multimedia dissemination methods, the opportunities for the older adults to access health information can be significantly increased, and their trust in health information can be strengthened.

#### 5.2.4 Promote the application of smart older adults care and intelligent devices to advance the scientific management of older adults health

With the development of technology, the application of smart older adults care and intelligent devices provides new avenues for health management among the older adults. This study emphasizes the central role of health information application in enhancing health awareness among the older adults. The government should vigorously promote the widespread use of smart health monitoring devices, such as heart rate monitors, blood glucose meters, and activity trackers, to help the older adults monitor their health status in real-time and detect potential health issues early. Additionally, the use of information technologies such as the Internet of Things (IoT), sensors, big data, and artificial intelligence should be promoted to collect, track, and monitor vital signs data from the older adults in real-time, transmitting the data to community service centers to form health records and provide personalized health management advice (41). The government should support the establishment of remote health monitoring systems and smart home systems to offer convenient health reminders and management services for the older adults. These technological tools can not only improve the scientific management of older adult health but also provide more precise and personalized health services, thereby effectively enhancing health awareness and improving health outcomes.

#### 5.2.5 Strengthen cross-sector collaboration and policy support to build a systematic guarantee for healthy aging

To ensure the effective implementation of the above strategies, the government should strengthen cross-sector collaboration and formulate and implement comprehensive healthy aging policies. These policies should cover multiple areas, including health education, eldercare services, and health insurance, to create a systematic support structure for enhancing health awareness among the older adults. The government should integrate social resources and promote collaboration among health departments, community service agencies, technology companies, and other stakeholders to develop and promote health management products and services for the older adults. Additionally, through policy incentives such as tax reductions and subsidies, the government should encourage enterprises to engage in innovative practices in older adult health management, fostering a collective focus and support across society for improving health awareness among the older adults.

Through these five strategies, the government and relevant institutions can systematically enhance the health awareness of the older adults, encouraging them to actively participate in health management. This not only contributes to the societal goal of “proactive health” among the older adults but also effectively reduces the burden on the healthcare system, making a positive contribution to sustainable societal development. This comprehensive strategy combines the enhancement of health awareness with modern technology, social resources, and policy support, demonstrating a forward-looking and scientific approach to addressing the challenges of aging.

## Data Availability

The original contributions presented in the study are included in the article/supplementary material, further inquiries can be directed to the corresponding author.
